# Effects of dietary levels of tapioca residue on growth performance and carcass characteristics in Hanwoo steers

**DOI:** 10.5713/ajas.18.0753

**Published:** 2019-01-04

**Authors:** Byung Ki Park, Dong Kyo Lee, Jun Sang Ahn, Joong Kook Park, Min Ji Kim, Gi Hwal Son, Jong Suh Shin

**Affiliations:** 1 Nonghyup Feed Co., LTD, Seoul 05398, Korea; 2 Hanwoo Research Institute, National Institute of Animal Science, RDA, Pyeongchang 25340, Korea; 3 College of Animal Life Sciences, Kangwon National University, Chuncheon 24341, Korea

**Keywords:** Blood Metabolite, Growth Performance, Hanwoo Steers, Meat Composition, Tapioca Residue

## Abstract

**Objective:**

This study was conducted to investigate the effects of dietary levels of tapioca residue on growth performance, carcass characteristics, and meat composition in Hanwoo steers.

**Methods:**

Twenty-eight steers were randomly assigned to one of four dietary groups; T0 (0% tapioca residue), T6.7 (6.7% tapioca residue), T9 (9% tapioca residue), and T12 (12% tapioca residue).

**Results:**

Supplementation with tapioca residue had no effect on overall growth performance. The concentration of plasma total cholesterol was higher in T6.7 than in other treatments (p<0.05). Dietary levels of tapioca residue did not affect carcass yield or the quality traits of Hanwoo steers. The lightness, redness, and yellowness of the *longissimus* muscle of Hanwoo steers were higher in T6.7 than in other treatments (p<0.05). Cohesiveness, gumminess, chewiness, and resilience were lower in T6.7 than in other treatments (p<0.05).

**Conclusion:**

The results of the present study indicate that supplementation with tapioca residue does not exert any negative effects on growth performance, carcass characteristics, and meat composition in Hanwoo steers. However, as the dietary level of tapioca residue increased, the intake of concentrate intake decreased, and tapioca supplementation greater than 6.7% did not substantially improved the marbling score.

## INTRODUCTION

In 2016, the production of concentrates for beef cattle was 4.5 million tons in Korea and reflects a steadily increasing trend in recent years. As corn is the most commonly used feed in concentrate formulations, corn imports have been steadily increasing [1]. In Korea, feed costs account for more than 60% of the total production cost of cattle, which increase concomitantly with increases in the usage rate of corn.

Tapioca which costs a mere 60% to 70% of the cost of corn can replace some of the energy (carbohydrates) of enriched raw materials, such as corn and wheat. As the nutrient content of tapioca (especially in terms of starch) is similar to that of corn, it is a valuable substitute for corn [2]. Despite this, the usage rate of tapioca for beef cattle feed remains between 2% and 4% in Korea.

It has been shown that replacing 30% of total corn usage with tapioca in growing pigs does not affect their productivity [3]. Furthermore, Enriquez and Ross [4] reported that replacing 50% of total corn usage with tapioca does not affect mortality, laying ability, and egg quality in laying hens.

In ruminants, the digestibility of tapioca is higher than that of sorghum and is similar to that of corn flakes [2]. The digestibility of tapioca starch in the rumen, small intestine, and colon were 94%, 5%, and 1%, respectively [5]. Muller et al [6] reported that tapioca starch was more digestible than general grains, but that it also required the addition of a nitrogen source for ruminal microbial protein synthesis owing to its low protein content (2.3% to 2.5%).

While previous studies [2,7] have been limited to the alternative status of tapioca in comparison to grains (corn, barley, oats, etc.), there have been few studies on the effects of tapioca residue supplementation on feed intake, growth performance, carcass characteristics, and meat quality in beef cattle. In particular, to the best of our knowledge, no studies have investigated changes in performance in response to dietary levels of tapioca residue in Hanwoo steers.

Therefore, the aim of this study was to investigate the effects of dietary levels of tapioca residue on growth performance, blood metabolites, carcass characteristics, and meat composition in the late fattening stages of Hanwoo steers.

## MATERIALS AND METHODS

The steers used in the present study were managed according to the scientific guidelines of the Animal Experiment Ethics Committee of Kangwon National University (No: KIACUC- 16-0010).

### Animals, treatments, and management

Twenty-eight Hanwoo steers (mean weight 619.8±63.8 kg, approximately 24 months of age) were randomly assigned to any one of four dietary treatments: T0 (0% tapioca residue), T6.7 (6.7% tapioca residue), T9 (9% tapioca residue), and T12 (12% tapioca residue). Steers were allotted by treatment group into four pens (5×10 m), each covered with 20 cm of sawdust.

Concentrate was provided three times daily (08:30, 13:00, and 17:00) using an automatic feeding system (SEOCHANG 65M/M, Seochang Co., Ltd., Cheonan, Korea) at approximately 1.8% of body weight (BW, as-fed basis) for the entire experimental period. Rice straw (dry matter 90.18%, crude protein 3.65%, ether extract 1.02%, crude fiber 34.19%, neutral detergent fiber 70.21%, acid detergent fiber 38.13%, crude ash 10.58%, calcium (Ca) 0.09%, and phosphorus [P] 0.05%) and water could be accessed freely. The formula percentages and nutrient contents of the concentrates are presented in Table 1. The chemical compositions of the experimental diets were analyzed by the standard methods of the AOAC [8].

### Feed intake, body weight, and blood characteristics

The average daily gain (ADG) was calculated by measuring BW at 10 am every 2 months. Feed intake was measured daily by measuring the leftover feed still present before the morning feeding. The feed conversion ratio (FCR) was calculated using dry matter intake (DMI) and ADG.

Blood samples (3 mL) for the analyses of blood metabolites were taken at 2-month intervals from the jugular vein of experimental animals using an 18-gauge needle and a blood collection tube (Vacutainer, Becton-Dickinson, Franklin Lakes, NJ, USA) coated with heparin. In addition, 3 mL of blood for blood corpuscle analyses was collected in another blood collection tube containing ethylenediaminetetraacetate.

Blood samples were stored in an ice box and transferred to the laboratory within 6 h of collection.

Blood samples were centrifuged at 1,250×*g* for 10 min to separate the plasma and analyzed using an automatic blood analyzer (Hitachi 7020, Hitachi Ltd., Tokyo, Japan). The analyses included glucose, total cholesterol (TC), albumin, total protein, triglyceride (TG), total bilirubin, blood urea nitrogen, gamma-glutamyl transpeptidase, glutamic oxaloacetic-transaminase, glutamic-pyruvic transaminase, non-esterified fatty acid (NEFA), creatinine, Ca, P, and magnesium (Mg).

Samples for the analyses of blood corpuscles were transferred to the laboratory and mixed using a roller mixer (1580R-Multi-purpose Centrifuge, LABOGENE, Bjarkesvej, Denmark). Red blood cell (RBC), red cell distribution width (RDW), reticulocytes (RETIC), white blood cell (WBC), hemoglobin (HGB), hematocrit (HCT), mean corpuscular volume (MCV), mean corpuscular hemoglobin (MCH), mean corpuscular hemoglobin concentration (MCHC), neutrophil count (NEU), eosinophil count (EO), basophil count (BA), lymphocyte count (LYM), leucocyte count with monocyte (MONO), and platelet count (PLT) were analyzed using a hematology analyzer (ProCyte Dx, IDEXX laboratories Inc., Westbrook, ME, USA).

### Carcass characteristics and meat composition of *longissimus* muscle

At the end of the experimental period (30 months of age), all animals were slaughtered at the local slaughterhouse to assess carcass yield and quality traits. Carcass evaluation was performed at the 13th rib section from the left side of each carcass by meat graders using the criteria provided by the Korean carcass grading system [9]. Meat quality traits were measured for marbling score, meat color, fat color, texture, and maturity. Yield traits were measured for carcass weight, back fat thickness, and rib eye area. The carcass yield index (YI) was calculated according to the following equation: YI = (68.184– [0.625×back fat thickness {mm}])+[0.130×rib eye area {cm^2^}]–[0.024×carcass weight {kg}])+3.23.

The chemical compositions of the *longissimus* muscle were measured according to the standard methods of the AOAC [8]. To measure the pH of meat, approximately 10 g of *longissimus* muscle was cut into small pieces and homogenized with 90 mL of distilled water (PolyTron PT-2500 E, Kinematica, Lucerne, Switzerland). The pH values were measured immediately after homogenization using a pH meter (Orion 230A, Thermo Fisher Scientific Inc., Waltham, MA, USA).

For the measurement of cooking loss, 1.0 cm-thick steaks were put in a polyethylene bag and heated in a water bath at 75°C for 40 min, and subsequently cooled at room temperature for 30 min. The percent cooking loss was determined by the difference in steak weights taken before and after cooking. Drip loss was measured as the weight loss during the suspension of a standardized (2×2×1 cm) sample sealed in a polyethylene bag at 4°C after 6 days of storage.

Water holding capacity (WHC) was measured according to the procedure of Hofmann and White [10]. Briefly, a 0.3 g sample of muscle was placed in a filter-press device and compressed for 5 min. After this process, WHC was calculated from duplicate samples as the ratio of the meat film area to the total area using an area-line meter (Super PLANIX-a, Tamaya Technics Inc., Tokyo, Japan).

Shear force values were determined using a Texture Analyzer (TA 1, LLOYD instruments LTD., Fareham, UK) with the following operating parameters: load cell, 50 kg; test and trigger speed, 50 mm/min; and trigger forces, 0.01 kgf.

Texture profile analyses were made by placing samples in a polyethylene bag and heating them to a constant temperature bath until the core temperature reached 75°C. After forming each *longissimus* muscle sample to 1×1×1 mm, the hardness, elasticity, cohesiveness, gumminess, and chewiness were measured using a texture analyzer equipped with a cylindrical probe of Ø35 mm (TA-XT plus, Stable Micro Systems Co., Ltd., London, UK). The samples were measured by pressing 80% of the sample height twice with pretest, test, and post-test speeds of 1 mm/s.

Meat color was measured using a colorimeter (Colormeter CR-300, Minolta Co., Osaka, Japan) immediately after removing the meat from the polyethylene bag. The color values of L* (lightness), a* (redness), and b* (yellowness) were repeatedly measured in the same manner. The standard white plate had a Y value = 93.60, an x value = 0.3134, and a y value = 0.3194.

The measurement of volatile basic nitrogen (VBN) was performed according to the method of Kim et al [11] using a Conway unit. Distilled water (90 mL) was added to *longissimus* muscle samples (10 g), was homogenized (PolyTron PT-2500 E, Kinematica, Switzerland), and subsequently centrifuged at 3,000×*g* for 10 min. The supernatant was filtered using filter paper and 0.01 N boric acid (1 mL). An indicator (0.066% methyl red:bromocresol green = 1:1) was added to the inner chamber of the Conway unit and the filtrate (1 mL), while 50% potassium carbonate (1 mL) was added to the outer chamber. The sealed Conway unit was maintained at 37°C for 2 h. The samples were titrated against 0.01 N sulfuric acid. The concentration of VBN was calculated as ammonia equivalents using the following equation: VBN (mg %) = (A–B)×F×28.014 ×100/S, where, A is the total amount of sulfuric acid (titrate sample [mL]), B is the total amount of sulfuric acid (titrate blank sample [mL]), F = 0.02 N standard index of sulfuric acid, and S is the sample weight (10 g).

The determination of 2-thiobarbituric acid reactive substances (TBARS) in the *longissimus* muscle was performed according to the methods of Witte et al [12]. Briefly, each sample (10 g) was added to 25 mL of 20% trichloroacetic acid (in 2 M phosphoric acid) and homogenized for 30 s. The samples were diluted with distilled water until the total amount of the homogenate was 50 mL and were then centrifuged (3,000×*g*, 4°C, 10 min). After centrifugation, the supernatant was filtered using filter paper and five milliliters of 0.005 mM TBARS was added to the filtrate (5 mL) and allowed to stand at room temperature for 15 h. The absorbance of the solution was measured at 530 nm using a UV/VIS spectrophotometer (M2e, Molecular Devices, Sunnyvale, CA, USA). TBARS was calculated according to the following equation: TBARS (mg of malondialdehyde/kg of sample) = (optical density [OD] of sample − OD of blank sample)×5.2.

### Statistical analyses

The least squares method was used to estimate the environmental effects on BW, ADG, blood characteristics, and carcass traits. The following linear model was used: y_ijkl_ = μ+TRT_i_+ β_1_X_1ij_+ β_2_X_2ik_+e_ijkl_, where μ = overall average; TRT_i_ = treatment effect (1 − 4); X_1_, X_2_ = the covariation of castration age and measurement month; β_1_, β_2_ = regression coefficient, and e_ijkl_ = random error effect.

The least squares method was also used to estimate environmental effects on feed intake and FCR. The following linear model was used: y_ij_ = μ+TRT_i_+e_ij_, where μ = overall average, TRT_i_ = treatment effect (1 − 4), and e_ij_ = random error effect.

The linear model was analyzed using SAS 9.1 [13] The package and variance analysis was performed using a Type III squared fit for unbalanced data among the four squares presented in the SAS/GLM analysis. The statistical significance of the differences between the least squares averages of the treatments were tested with the following null hypothesis at a significance level of 5%: Ho: lease squares means (LSM) (i) = LSM (j), where LSM (i(j)) is the least squares average of the I (j) effects (I ≠ j).

## RESULTS

### Growth performance and blood characteristics

The effects of the dietary level of tapioca residue on BW gain, feed intake, and FCR of late fattening Hanwoo steers are shown in Table 2. The difference in initial BWs between the highest and lowest groups was 10.86 kg; however, this difference was not statistically significant. The final BWs and ADG values were slightly but not significantly higher in T6.7 than in the other treatments.

The concentrate intake and DMI tended to decrease with increasing dietary levels of tapioca residue. The FCR was slightly but not significantly lower in T6.7 than in the other treatments.

The effects of the dietary level of tapioca residue on blood metabolites and blood corpuscles in late fattening Hanwoo steers are shown in Tables 3 and 4. The concentration of TC was higher in T6.7 than in the other treatments (p<0.05). The concentration of TGs was the lowest in T6.7, while the highest concentration was found in T12 (p<0.05). The concentration of Mg was the lowest in T12 (p<0.05), while the concentrations observed in other treatment groups was similar. The concentration of NEFA was higher in T9 than in T0 and T6.7 (p<0.05). Supplementation with tapioca residue exerted no effects on RBC, HCT, HGB, MCV, MCHC, RDW, RETIC, WBC, NEU, LYM, MONO, EO, BA, and PLT in late fattening Hanwoo steers.

### Carcass characteristics and meat composition of the *longissimus* muscle

The effects of the dietary level of tapioca residue on carcass characteristics in Hanwoo steers are shown in Table 5. The dietary level of tapioca residue did not affect carcass weight, back fat thickness, rib eye area, or yield index in Hanwoo steers. Furthermore, the dietary level of tapioca residue did not affect meat color, fat color, texture, or the maturity of Hanwoo steers. The marbling score was slightly but not significantly higher in T6.7 than in other treatments. Meat color and maturity were similar between the treatments.

The effects of the dietary level of tapioca residue on the meat composition in the *longissimus* muscle of Hanwoo steers are shown in Table 6. The content of ether extract in the *longissimus* muscle was slightly but not significantly higher in T6.7, T9.0, and T12 than in T0. The dietary level of tapioca residue did not affect the content of crude protein or the pH value of the *longissimus* muscle of Hanwoo steers. The lightness, redness (p<0.05), and yellowness (p<0.05) of the *longissimus* muscle of Hanwoo steers were higher in T6.7 than in other treatments. The content of VBN tended to increase with increasing dietary levels of tapioca residue; however, the differences were not statistically significant. Hardness, springiness, cohesiveness, gumminess, chewiness, and resilience were lower in T6.7 than in other treatments (p<0.05).

## DISCUSSION

### Growth performance and blood characteristics

In the present study, although the difference was not statistically significant, concentrate intake decreased as the dietary level of tapioca residue increased during the late fattening period (Table 2). A similar finding was reported by Zinn and DePeters [7], wherein feed intake and efficiency were lower in the treatment group that received 30% replacement than in the 15% replacement treatment group when replacing corn flakes with tapioca. In addition, Garcia and Dale [14] reported that palatability and feed intake tended to decrease as the dietary level of tapioca increased, in agreement with the results of the present study.

In addition, the results of the present study show that as the dietary level of tapioca residue increased, feed intake tended to decrease slightly. However, tapioca residue did not affect ADG and FCR. This finding is in agreement with the findings of other studies, which have also reported that the digestibility of tapioca is comparable to corn flakes [7,15].

We found no effects of supplementation with tapioca residue on ADG and FCR in late fattening Hanwoo steers. Likewise, previous studies have shown that substituting corn, barley, and sorghum with tapioca did not affect the ADG or FCR of beef cattle [7,15].

However, our results differ from previous studies, which found that the substitution of corn and barley with tapioca residue enhanced productivity measures such as ADG and FCR in buffalo calves [16] and dairy cattle [17]. The differences observed between studies could be explained by differences in the types of experimental diets, as well as the quality and processing methods of the tapioca residue. Furthermore, Adebowale et al [18] found that the chemical composition of tapioca varied as a result of both genetic variation and processing (roasting) methods. They also reported that the dietary composition of tapioca was 7.20% to 10.50% moisture, 1.88% to 2.75% sugar, 86.96% to 94.75% starch, 89.79% to 97.34% carbohydrate, 0.23% to 0.26% protein, and 0.12% to 0.25% fat.

The concentration of plasma cholesterol in beef cattle is generally correlated with the fat content (degree of marbling) of the carcass, and thus the high concentration of plasma cholesterol in the T6.7 group (6.7% tapioca residue) directly affected the marbling score (Table 5). Wheeler et al [19] reported that the concentration of serum cholesterol in crossbred species with higher contents of carcass fat was higher than that of Chianina species, and that the carcass fat and lipid contents of the *longissimus* muscle were correlated with the concentration of serum cholesterol (values of 0.71 and 0.63, respectively). In addition, the concentration of plasma cholesterol was higher in the T6.7 group, which also had a slightly higher feed energy (total digestible nutrients [TDN] and ether extract) than the other treatment groups. This finding is similar to the finding of Arave et al [20], who reported that serum cholesterol concentrations increased as energy intake increased.

The concentration of plasma NEFA has been used as an indicator of nutritional status and energy balance and has been reported to increase when either feed intake is reduced or energy is lacking [21]. In the present study, the NEFA concentration was higher in the T9 and T12 groups than in the T6.7 group, which may be related to the decrease in feed intake observed at the higher levels of tapioca residue supplementation (Table 2). The low NEFA concentration observed in the T6.7 group suggests the inhibition of body fat degradation [22] and the effective synthesis of fatty acids from NEFA (a precursor of lipid synthesis) by insulin. In addition, it is possible that low NEFA-likely owing to lipid synthesis by insulin is related to the high marbling score observed in the T6.7 group (Table 5).

Blood corpuscle parameters (e.g., WBC, RBC, HGB, HCT, MCV, and MCH) are the main blood components of hematopoiesis, and are important in the diagnosis, treatment, and assessment of the physiological status of ruminants [23]. We found no differences in blood corpuscle parameters between the treatment groups supplemented with tapioca residue, and all parameters were within their normal ranges.

### Carcass characteristics and meat composition of the *longissimus* muscle

In the present study, supplementation with tapioca residue had little effect on the back fat thickness, rib eye area, and marbling scores of Hanwoo steers. This may be owing to similar levels of feed intake and ADG values (Table 2), which reflect the similar energy (TDN) and crude protein contents (Table 1) of concentrates between the treatments.

The Korea Institute for Animal Products Quality Evaluation (KAPE) [24] reported that a total of 2,022,791 Hanwoo steers were slaughtered from 2012 to 2016 in Korea, and that the average carcass weight, back fat thickness, rib eye area, and marbling scores were 425.8 kg, 13.2 mm, 90.4 cm^2^, and 5.5, respectively. The results of KAPE are different from the results of the present study, which may be a result of differences in the castration and slaughter ages. In most Hanwoo farms in Korea, calves are castrated at 6 to 8 months of age and are slaughtered at 31 to 32 months of age. However, in the present study, the castration and slaughter ages were 14 and 30 months, respectively. Because of the delayed castration age and younger slaughter age in our study, marbling scores were slightly lower; however, carcass weights and rib-eye areas were higher in comparison to the average results reported by KAPE [24].

Lee et al [25] reported that moisture and ether extract contents were influenced by the proportion of marbling. In the present study, ether extract contents in the *longissimus* muscle tended to be higher in the T6.7 group, which also exhibited higher marbling. This finding is concurrent with that of Chin et al [26], who found that the contents of ether extract were increased in proportion to marbling.

Wulf and Page [27] reported that the normal pH of beef is less than 5.75. In the present study, the pH of the *longissimus* muscle was normal in all treatments, indicating that supplementation with tapioca residue had no effect on the pH of the *longissimus* muscle.

In addition to marbling, meat color is an important factor in meat quality grading and consumer preferences, and it has been reported that brightness, redness, and yellowness all increased in proportion to marbling [25]. In agreement with previously reported results, the lightness, redness, and yellowness of meat were higher in the T6.7 group the present study, which is related to the higher degree of marbling observed in this group. However, redness and yellowness tended to be lower in the T9 and T12 groups in comparison to T0 (0% tapioca residue), despite the differences in the marbling score. This finding may be a result of the decreased proportion of corn, which contains carotenoid-type pigments such as chlorophyll or xanthophyll [28].

In the present study, the WHC of *longissimus* muscle was not affected by the different marbling scores, which are also related to tapioca supplementation levels. This is similar to the findings of Lee et al [29] and Chin et al [26], wherein the marbling scores and the quality grades of meat did not affect water holding capacity.

We found that the shear force and other physical properties (elasticity, cohesiveness, stickiness, and chewiness) of the *longissimus* muscle tended to be lower as supplementation with tapioca residue increased, which was also related to the observed increase in marbling scores. Lee et al [29] reported that the shear force decreased significantly from 8.29 to 2.83 kg as meat grade increased from 3 to 1^++^, while the results of other previous studies [30,31] are also in agreement with the results of the present study.

In conclusion, supplementing the diets of Hanwoo steers with tapioca residue had no negative effects on growth performance, carcass characteristics, and meat composition. However, as the dietary level of tapioca residue increased, the concentrate intake decreased, and thus supplementing diets with more than 6.7% tapioca hardly improved the marbling score.

## Figures and Tables

**Table 1:** Ingredient and chemical compositions of the experimental diets of Hanwoo steers

Item	Treatments			

	T0	T6.7	T9.0	T12.0
	------------- Ingredient composition (%) -----------			
Corn grain	37.9	34.0	34.1	34.0
Wheat grain	10.0	10.0	10.0	10.0
Cane molasses	5.0	5.0	5.0	5.0
Wheat flour	2.0	2.0	2.0	2.0
Tapioca residue	-	6.7	9.0	12.0
Soybean meal	-	-	-	2.0
Wheat bran	5.6	1.0	1.0	1.0
Corn gluten feed	20.0	20.0	17.1	11.7
Rapeseed meal	2.2	3.7	5.0	5.0
Palm kernel meal	9.0	9.0	6.0	6.0
Lupin	2.5	2.5	2.5	2.5
Rice polishing	1.0	1.0	1.0	1.0
Marblelist (feed additive)	1.0	1.0	1.0	1.0
Emulsifier	0.1	0.1	0.1	0.1
Vitamin premix1)	0.1	0.1	0.1	0.1
Mineral premix2)	0.1	0.1	0.1	0.1
Sodium bicarbonate	0.5	0.5	0.5	0.5
	---------- Chemical composition (%) -------------			
Dry matter	88.30	88.23	88.14	87.98
Crude protein	12.21	12.23	12.18	12.26
Ether extract	3.58	3.45	3.37	3.32
Crude fiber	5.75	6.42	6.33	6.37
Crude ash	6.17	5.92	5.98	5.89
Ca	0.91	0.75	0.75	0.75
P	0.41	0.39	0.38	0.37
TDN	73.44	74.12	73.80	73.67

TDN, total digestible nutrients (calculated value).

1) Vitamin premix provided the following quantities of vitamins per kilogram of diet: vitamin A, 10,000 IU; vitamin D_3_, 1,500 IU; vitamin E, 25 IU.

2) Mineral premix provided the following quantities of minerals per kilogram of diet: Fe, 50 mg; Cu, 7mg; Zn, 30 mg; Mn, 24 mg; I, 0.6 mg; Co, 0.15 mg; Se, 0.15 mg.

**Table 2:** Effects of dietary levels of tapioca residue on growth performance in late fattening Hanwoo steers

Item	Treatments				SEM	Pr>F

	T0	T6.7	T9.0	T12.0		
Body weight (kg)						
Initial BW	621.14	615.43	626.29	616.43	25.52	0.99
Final BW	744.29	748.57	744.29	734.14	31.05	0.99
Average daily gain	0.68	0.74	0.65	0.65	0.01	0.73
Intake (DM, kg)						
Concentrate	10.31	10.17	9.63	9.56	0.46	0.85
Rice straw	1.00	1.00	0.72	1.00	0.16	0.96
Dry matter	11.31	11.17	10.35	10.56	0.53	0.78
Feed conversion ratio	16.63	15.10	15.92	16.25	1.22	0.92

SEM, standard error of mean; BW, body weight; DM, dry matter.

**Table 3:** Effects of dietary levels of tapioca residue on blood metabolites in late fattening Hanwoo steers

Item	Treatments				SEM	Pr>F

	T0	T6.7	T9.0	T12.0		
Total protein (g/dL)	7.40	7.43	7.21	7.43	0.12	0.45
Albumin (g/dL)	3.91	3.90	3.90	3.77	0.05	0.18
Total bilirubin (mg/dL)	0.59	0.54	0.59	0.47	0.05	0.39
AST (IU/L)	75.51	72.04	73.03	67.81	3.81	0.56
ALT (IU/L)	21.50	21.10	19.73	19.09	0.94	0.25
Ca (mg/dL)	9.66	9.59	9.59	9.30	0.13	0.25
GGT (mg/dL)	29.27	23.50	24.69	33.96	3.58	0.18
Glucose (mg/dL)	55.97	56.59	55.17	56.19	1.42	0.91
Cholesterol (mg/dL)	124.57^b^	158.06^a^	130.84^b^	119.39^b^	7.72	0.01
Phosphorus (mg/dL)	6.96	6.83	6.89	6.79	0.18	0.92
BUN (mg/dL)	15.63	15.53	16.34	14.26	0.63	0.16
Creatinine (mg/dL)	1.29	1.27	1.40	1.33	0.39	0.12
NEFA (uEq/L)	223.03^b^	219.59^b^	270.20^a^	237.86^ab^	11.67	0.02

SEM, standard error of mean; AST, aspartate aminotransferase; ALT, alanine aminotransferase; GGT, gamma glutamyl transferase; BUN, blood urea nitrogen; NEFA, non-esterified fatty acid.

a,b Mean values followed by different letters in the same row are significantly different (p<0.05).

**Table 4:** Effects of dietary levels of tapioca residue on blood corpuscle parameters in late fattening Hanwoo steers

Item	Treatments				SEM	Pr>F

	T0	T6.7	T9.0	T12.0		
RBC (M/μL)	7.83	8.04	7.78	7.32	0.21	0.14
HCT (%)	37.83	39.03	38.13	36.47	1.35	0.61
HGB (g/dL)	12.74	12.91	12.72	12.18	0.41	0.62
MCV (fL)	48.42	48.60	49.03	49.94	1.46	0.88
MCH (pg)	16.29	16.10	16.35	16.70	0.42	0.78
MCHC (g/dL)	33.73	33.21	33.36	33.49	0.44	0.86
RDW (%)	29.08	29.64	28.38	27.19	0.77	0.16
RETIC (%)	6.90	7.29	7.34	6.84	0.63	0.92
WBC (K/μL)	9.11	9.63	9.11	8.73	0.53	0.69
NEU (K/μL)	3.02	2.94	2.67	2.91	0.23	0.72
LYM (K/μL)	4.70	5.16	5.18	4.41	0.33	0.31
MONO (K/μL)	0.47	0.46	0.39	0.44	0.03	0.45
EOS (K/μL)	0.83	1.06	0.85	1.03	0.12	0.41
BASO (K/μL)	0.03	0.02	0.02	0.02	0.01	0.26
PLT (K/μL)	214.60	188.79	190.81	213.67	17.22	0.58

SEM, standard error of mean; RBC, red blood cell; HCT, hematocrit; HGB, hemoglobin; MCV, mean corpuscular volume; MCH, mean corpuscular haemoglobin; MCHC, mean corpuscular hemoglobin concentration; RDW, red cell distribution width; RETIC, reticulocytes; WBC, white blood cell; NEU, neutrophil; LYM, lymphocyte count; MONO, monocyte; EOS, eosinophil; BASO, basophil; PLT, platelet count.

**Table 5:** Effects of dietary levels of tapioca residue on carcass characteristics in Hanwoo steers

Item	Treatments				SEM	Pr>F

	T0	T6.7	T9.0	T12.0		
Yield traits1)						
Carcass weight (kg)	446.71	449.57	443.57	449.14	20.54	0.99
Back fat thickness (mm)	14.29	14.29	12.71	14.57	1.84	0.89
Rib eye area(cm^2^)	91.00	93.57	90.71	92.43	2.52	0.84
Yield index	63.59	63.86	64.61	63.54	1.24	0.92
Quality traits2)						
Marbling score	3.00	4.57	3.57	3.57	0.64	0.39
Meat color	5.00	5.00	5.00	5.14	0.07	0.41
Texture	1.86	1.29	1.43	1.71	0.18	0.13
Maturity	2.00	2.00	2.00	2.00	-	-

SEM, standard error of mean.

1) Area and back fat thickness were measured from the longissimus muscle taken at the 13th rib. Yield index was calculated using the following equation: (68.184 – [0.625×back fat thickness {mm}]+[0.130×rib eye area {cm^2^}]–[0.024×dressed weight amount {kg}])+3.23.

2) Grading ranges are 1 to 9 for marbling score, where higher numbers indicate better quality (1 = devoid, 9 = abundant); meat color (1 = bright red, 7 = dark red); texture (1 = soft, 3 = firm); and maturity (1 = youthful, 9 = mature).

**Table 6:** Effects of dietary levels of tapioca residue on meat composition in the longissimus muscles of Hanwoo steers

Item	Treatments				SEM	Pr>F

	T0	T6.7	T9.0	T12.0		
Moisture (%)	67.17	63.30	65.20	66.26	1.20	0.17
Ether extract (%)	11.47	17.03	14.34	14.38	1.60	0.15
Crude protein (%)	22.05	20.17	20.06	19.57	0.63	0.18
pH	5.59	5.63	5.61	5.62	0.02	0.41
CIE L^*^	42.79	44.03	42.32	42.29	0.89	0.58
CIE a^*^	20.77^ab^	21.28^a^	19.07^c^	19.19^b^^c^	0.53	0.02
CIE b^*^	10.38^a^	10.81^a^	9.34^b^	9.30^b^	0.30	0.01
Cooking loss (%)	31.26^b^	31.50^ab^	30.70^b^	32.86^a^	0.44	0.04
Drip loss (%)	6.52	6.39	4.98	5.52	0.51	0.15
WHC (%)	45.14	42.31	44.01	43.50	1.10	0.40
WBSF (kg)	5.84^ab^	4.75^b^	4.71^b^	6.48^a^	0.43	0.03
VBN (mg %)	6.20	6.26	6.64	6.74	0.19	0.19
TBARS (mg MA/kg)	0.34^b^	0.33^b^	0.49^a^	0.47^ab^	0.04	0.03
Hardness (kg)	7.47	5.86	6.12	6.65	0.41	0.07
Springiness	0.52	0.50	0.50	0.52	0.01	0.41
Cohesiveness	0.43^ab^	0.38^b^	0.43^ab^	0.46^a^	0.02	0.05
Gumminess	3.27^a^	2.24^b^	2.62^ab^	3.05^a^	0.22	0.02
Chewiness (kg)	1.67^a^	1.11^b^	1.32^ab^	1.62^a^	0.12	0.01
Resilience (mm)	0.24^a^	0.20^b^	0.22^ab^	0.23^a^	0.01	0.01

SEM, standard error of mean; CIE, Commission Internationale de l’Eclairage; WHC, water holding capacity; WBSF, Warner-Bratzler shear force; VBN, volatile basic nitrogen; TBARS, 2-Thiobarbituric acid reactive substances.

a,b Mean values followed by different letters in the same row are significantly different (p<0.05).
